# The Dynll1-Cox4i1 Complex Regulates Intracellular Pathogen Clearance via Release of Mitochondrial Reactive Oxygen Species

**DOI:** 10.1128/IAI.00738-19

**Published:** 2020-03-23

**Authors:** Jiangbei Yuan, Zihan Zheng, Liting Wang, Haiying Ran, Xiangyu Tang, Xiaodong Xie, Fei Li, Fang Liu, Xiaoyang Wang, Jiale Zhang, Junying Zhang, Yi Huang, Xuefeng Xia, Ying Wan

**Affiliations:** a School of Pharmaceutical Sciences and Innovative Drug Research Center, Chongqing University, Chongqing, China; b Biomedical Analysis Center, Army Medical University, Chongqing, China; c Chongqing Key Laboratory of Cytomics, Chongqing, China; Yale University School of Medicine

**Keywords:** Dynll1-Cox4i1 complex, *Listeria monocytogenes*, reactive oxygen species, membrane proteins, proteomics

## Abstract

Cellular membrane proteins are a critical part of the host defense mechanisms against infection and intracellular survival of Listeria monocytogenes. The complex spatiotemporal regulation of bacterial infection by various membrane proteins has been challenging to study. Here, using mass spectrometry analyses, we depicted the dynamic expression landscape of membrane proteins upon L. monocytogenes infection in dendritic cells. We showed that Dynein light chain 1 (Dynll1) formed a persistent complex with the mitochondrial cytochrome oxidase Cox4i1, which is disturbed by pathogen insult. We discovered that the dissociation of the Dynll1-Cox4i1 complex is required for the release of mitochondrial reactive oxygen species and serves as a regulator of intracellular proliferation of Listeria monocytogenes. Our study shows that Dynll1 is an inhibitor of mitochondrial reactive oxygen species and can serve as a potential molecular drug target for antibacterial treatment.

## Abstract (precis)

Cellular membrane proteins are a critical part of the host defense mechanisms against infection and intracellular survival of Listeria monocytogenes. The complex spatiotemporal regulation of bacterial infection by various membrane proteins has been challenging to study. Here, using mass spectrometry analyses, we depicted the dynamic expression landscape of membrane proteins upon L. monocytogenes infection in dendritic cells. We showed that Dynein light chain 1 (Dynll1) formed a persistent complex with the mitochondrial cytochrome oxidase Cox4i1, which is disturbed by pathogen insult.

## INTRODUCTION

The cellular membranes of phagocytic cells are highly complex interfaces that perform a broad spectrum of internal and external environment-sensing reactions. These reactions span wide and distinct spatiotemporal ranges, which has made it difficult to identify and functionally characterize membrane proteins (MPs) (1, 2). This difficulty is particularly evident in the context of bacterial infection, during which MPs located on many different types of phagocyte cellular membranes have been shown to play critical roles (3). For instance, classical cell membrane-localized pattern recognition receptors (PRRs) have evolved to sense the presence of bacterial components, such as lipopolysaccharides, flagellins, and lipids (4, 5). Ligation of antigens to these receptors triggers rapid movement of molecules such as Myd88 and Irak4 to and from the membrane to activate signaling cascades (6, 7). Other internal membrane sensors, such as stimulator of interferon genes (STING) (8), cGAS (9), and mTOR (10), can be activated when bacterial components are detected. Activation of these external and internal sensors is dependent upon dramatic alterations in MP distribution in multiple organelles.

Once bacteria are sensed, a wide range of innate cellular responses is initiated to clear the bacteria. The first line of defense is the process of direct phagocytosis, during which the plasma membrane undergoes an MP-controlled involution to take up bacteria (11). Degradation/sequestration of the cargo is regulated by phagosome-bound MPs via a complex and intricate set of processes (12). Several components of the autophagosome influx system have been reported to participate in the cargo degradation process (13, 14). Coating and pH modification of the phagosomes are regulated by membrane-bound vesicular ATP pumps and small GTP hydrolases (GTPases) that render the environment inhospitable for bacteria (3). We and others have previously shown that GTPase Rab32 trafficking is critically involved in the bacterial containment process via regulation of biogenesis of lysosome-related organelle complexes (BLOCs) (15–17). Collectively, these phagocytic cell defenses present substantial challenges that allow for efficient elimination of most prospective bacteria.

To overcome the defense systems of phagocytic cells, successful bacteria have evolved countermeasures to modify and evade MPs. These countermeasures are most commonly seen in infection of Listeria monocytogenes, Salmonella enterica serovar Typhimurium, and Shigella flexneri, which have gained the ability to reside in host immune cells designed to eliminate them (18–20). These mechanisms include uniquely secreted proteins that can cleave Rab32 and inhibit BLOC function (*S*. Typhimurium GtgE) and pore-forming enzymes that can rupture the phagosome *(L. monocytogenes* listeriolycin O [LLO]) (21). Once these bacteria escape from the phagosome, they can hijack the host cytoskeleton to cross through cell membranes (for example, L. monocytogenes uses its surface actin-assembly inducing protein ActA, and S. flexneri uses LcsA, a surface autotransporter protein) (22). It has also been suggested that intracellular bacteria may disrupt the normal phagocytosis process to increase nutrient load in their favor. It is currently unknown whether the host cells possess additional mechanisms to counteract these bacterial offensive strategies.

In this study, we performed a comprehensive proteomics search to delineate differences in the expression profiles of MPs in dendritic cells (DCs) over the course of L. monocytogenes infection. From this characterization, we aim to identify potential mechanisms by which DCs control the intracellular proliferation of L. monocytogenes.

## RESULTS

### The membrane proteomes of dendritic cells are altered by *L. monocytogenes* infection.

**FIG 1:**
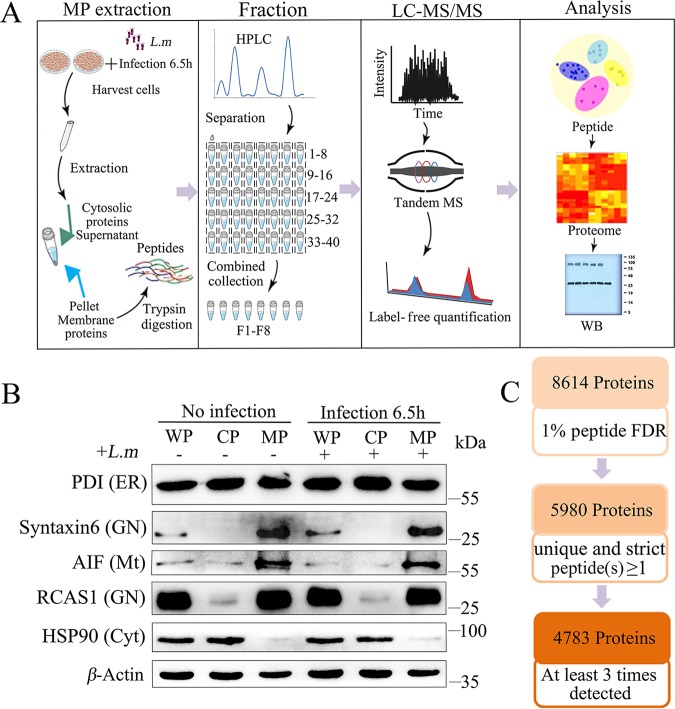
Membrane proteomic preanalysis in DCs infected with L. monocytogenes. (A) General workflow of MS-based quantitative proteomic and bioinformatics analyses. MP, membrane protein; HPLC, high-performance liquid chromatography; LC-MS, liquid chromatography-mass spectrometry; WB, Western blotting. (B) Western blotting of membrane protein extractions using known organelle marker proteins: protein disulfide isomerase (PDI) for the endoplasmic reticulum, apoptosis-inducing factor 1 (AIF) for mitochondria, Syntaxin-6 (Stx6) and Receptor-binding cancer antigen expressed on SiSo cells (RCAS1) for the Golgi apparatus, and total HSP90 protein (HSP90) for the cytoplasm. WP, whole protein; CP, cytoplasmic protein; MP, membrane protein. (C) Workflow for data filtering and processing of membrane proteins using label-free quantitative mass spectrometry. FDR, false detection rate.

To analyze the membrane proteomes of DCs, we first optimized a protocol for isolating only MPs, while avoiding cytoplasmic and other types of organelle contamination (Fig. 1A). Briefly, cells were first passed through the proprietary filter (Invent Biotechnologies) in a zigzag manner when a high-speed centrifugal force was applied, resulting in the cell lysate obtained. Membrane proteins and cytosol proteins were further separated from the cell lysate by subsequent differential centrifugation. We tested the efficacy of this protocol by checking the expression of established organelle markers using Western blotting. Consistent with our expectations, the Golgi compartment-localized Rcas1 and the *trans*-Golgi SNARE protein syntaxin-6 were strongly enriched in the MP portion of cell lysates, whereas the cytosolic chaperone Hsp90 was notably excluded (Fig. 1B). We then performed high-performance liquid chromatography-mass spectrometry (HPLC-MS) of the MPs by subdividing each sample into 40 fractions based on elution time; the fractions were then merged into eight portions for analysis (Fig. 1A). From this procedure, we recovered a total of 8,614 unique proteins at a peptide false discovery rate (FDR) cutoff of 1% in six experiments (three no-infection and three infected DC fractions), 4,783 of which were shared among at least three experiments (Fig. 1C and see Table S1 in the supplemental material). Wild-type (WT; no infection) or infected DC correlations between protein expression profiles across different samples showed a Spearman’s correlation coefficient that exceeded 0.8 under these cutoff parameters (Fig. S1A). Notably, our strategy was also able to recover MPs from all major intracellular organelles (Table S1). Principal component analysis (PCA) proved that the infected and WT DCs (no infection) were completely separated, and the three biological repeats of each DC type appeared to be well clustered (Fig. S1B). The distribution of the logarithmic fold changes of proteins is presented in a histogram (Fig. S1C). The global MS data of MPs are displayed using a heat map (Fig. S1D). Beyond changes in host proteins, we also found a large number of *Listeria* proteins in the infected DCs, such as actin-assembly inducing protein (ActA; Lmrg_02626) and listeriolysin O (LLO; Lmrg_02624) (Table S2), confirming that our method was sensitive to the biological context of the assay. Collectively, these results supported the robustness of our fractionation and MS approaches.

**FIG 2:**
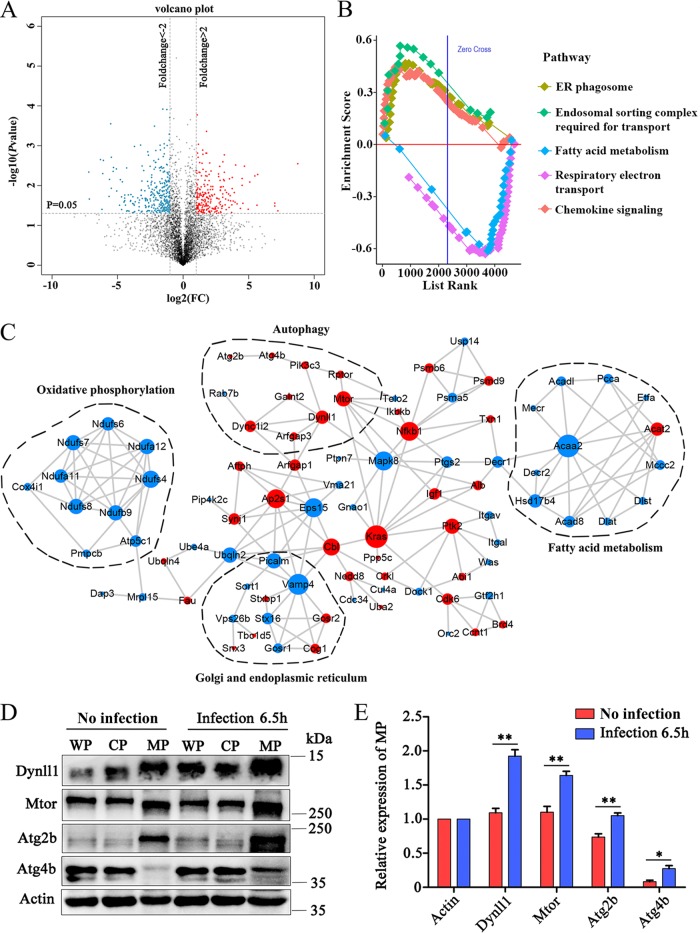
The proteomic landscape and biological validation of membrane proteins using label-free quantitative MS. (A) Differentially expressed proteins of no-infection DCs and DCs infected with L. monocytogenes are shown in a volcano plot. The mean ratios of three biological repeats (infected cells at 6.5 h versus no infection) were plotted on a log_2_ scale (*x* axis) against the corresponding –log_10_ *P* value (*y* axis). Proteins representing fold changes (FC) of >2 or <0.5 (*P* < 0.05) were considered up- or downregulated and are indicated in red and blue, respectively. (B) Pathway analyses identified proteins involved in major biological pathways. *P* < 0.01 was considered significant. (C) Network analysis of differentially expressed proteins. Red and blue show increased and decreased protein expressions, respectively. Sizes of nodes are depicted based on the numbers of interacting neighbors. (D and E) Detection and quantification of Western blot signals of four autophagy-related proteins in no-infection and infected DCs. WP, whole protein; CP, cytoplasm protein; MP, membrane protein (*n* = three samples were analyzed for each case). Two-way ANOVA with Tukey-Kramer test was used to measure significance. All data are shown as means ± the SEM. *, *P* < 0.05; **, *P* < 0.01; ***, *P* < 0.001.

After optimization, we next quantified the changes in the membrane proteome upon infection of DCs with L. monocytogenes. Differentially expressed proteins were depicted using a volcano plot (Fig. 2A); 223 proteins showed a 2-fold increase and 327 proteins exhibited a 2-fold decrease in expression (*P* < 0.05; Table S1). Up- or downregulated proteins were analyzed using gene ontology (GO) term enrichment analysis based on the categories of cellular component, biological process, and molecular function (Fig. S1E). Pathway analyses of MPs expressed postinfection showed significant enrichment of factors involved in endosomal sorting and phagosome function, which were consistent with typical DC functions (Fig. 2B) (23–25). We also observed significantly diminished expression of proteins involved in the pathways regulating the metabolic capacity of cells, such as fatty acid metabolism and respiratory electron transport, which was consistent with the effects of infection-induced stress on DCs (Fig. 2B) (26–28). These results were further supported by induced network analyses, in which proteins from each of the most-altered pathways were mapped based on the existing protein-protein interaction databases (Fig. 2C). Interestingly, both GO and network analyses showed that the autophagy pathway-related MPs showed prominent upregulation in expression. Previous reports have shown that the mTORc1 complex acts as an internal membrane sensor by localizing in lysosomal membranes under conditions of various stresses (29–32). Western blotting of whole protein, MP, and cytosolic fractions of DCs showed increased membrane localization of mTOR in infected cells, even though the total protein content did not significantly vary between WT (no infection) and infected DCs (Fig. 2D and E; see also Fig S2). In addition, other autophagy-related proteins—Atg2b, Atg4b, and Dynll1—also showed similar expression patterns, suggesting that membrane recruitment of proteins to the autophagosome may be important for the DC response to infection (Fig. 2D and E; see also Fig. S2). Among them, the expression of Dynll1 in MP increased upon infection was the most significant of the four autophagy-related proteins (Fig. 2E). These data were consistent with our mass spectral data (Fig. S3A). Immunofluorescence analyses showed increased overall fluorescence intensity of Dynll1 and increased intensity of Atg2b on the peripheral membrane of the DCs; however, Atg4b and mTOR did not show any significant change in the overall fluorescence intensity (see Fig. S3B and C in the supplemental material).

### Dynll1 functions distinctly from traditional autophagosomal proteins.

**FIG 3:**
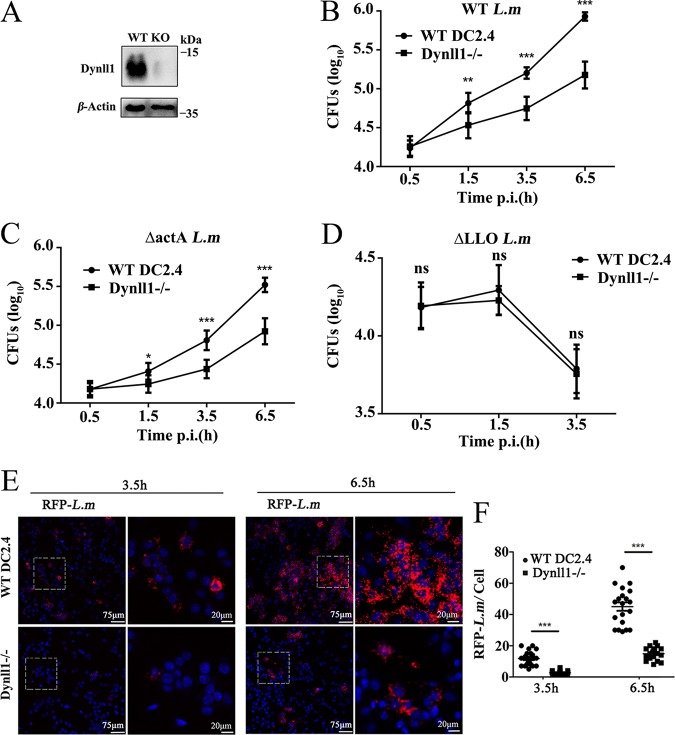
Dynll1 promotes intracellular viability of L. monocytogenes in dendritic cells. (A and B) CFU recovered from WT and Dynll1 knockout DCs after infection with L. monocytogenes (*n* = 8). KO, knockout; DCs, dendritic cells. (C) CFU recovered from WT and Dynll1 KO DCs infected with ActA-deficient L. monocytogenes (the ActA mutant has limited intra- and intercellular mobility) (*n* = 8). (D) CFU recovered from WT and Dynll1 KO DCs infected with LLO-deficient L. monocytogenes (LLO deficiency limits the escape of bacteria to the cytoplasm) (*n* = 8). LLO, listeriolysin O. (E and F) Visualization by immunofluorescence microscopy and quantification of RFP-tagged L. monocytogenes, in which the RFP tag is expressed under the *actA* promoter, in WT and Dynll1 knockout dendritic cells infected for 3.5 and 6.5 h. Each dot in panel F represents the number of RFP-tagged L. monocytogenes/cell. RFP, red fluorescent protein. We applied two-way ANOVA with Tukey-Kramer tests. All data are shown as means ± the SEM. *, *P* < 0.05; **, *P* < 0.01; ***, *P* < 0.001.

Next, we functionally characterized the proteins belonging to the autophagy-related pathway. Because the autophagosomal complex is important for preventing pathogen escape (18, 33), we anticipated the upregulated proteins in the pathway to be critical for controlling intracellular bacterial content. Consistent with this hypothesis, knockdown of *Atg2b* and *Atg4b* led to an increase in the amounts of viable L. monocytogenes (Fig. S4A and B). However, knockout (KO) of Dynll1 actually led to a decrease in the number of CFU (Fig. 3A and B). It was reported that Dynll1 interacted with BECN1 (Beclin1) and recruited BCL211 (also known as BIM) to microtubules, thereby inhibiting autophagy. Under starvation conditions, the Dynll1-Beclin1 complex dissociated, thereby ameliorating autophagy inhibition (34, 35). These studies suggested that Dynll1 may be a multifunctional small molecule involved in autophagy. In order to verify the function of Dynll1 upon L. monocytogenes infection, various mutant L. monocytogenes strains were used. This effect of Dynll1 was also observed in ActA-deficient strains of L. monocytogenes, in which the pathogen continues to persist in the cytoplasm after lysosome escape instead of propagating into neighboring cells (Fig. 3C), suggesting that Dynll1 served some function that was independent of intercellular propagation. Interestingly, Dynll1 deficiency had no impact on L. monocytogenes cells that lacked the phagosome-rupturing enzyme (LLO), which is incapable of vacuole escape, suggesting that Dynll1 may mostly function in response to intracellular proliferation (Fig. 3D). In addition, lower numbers of lysosome-escaped bacteria (containing reporter-tagged *actA*) were observed in Dynll1^–/–^ mutants than in WT cells (Fig. 3E and F); this result further supports that the Dynll1^–/–^ mutant affects the intracellular proliferation of *Listeria*.

In order to rule out the effect of host cell death on CFU experimental perturbations, we further performed apoptosis assays and live cell counts on WT and Dynll1^–/–^ mutant during the *Listeria* infection at the time points we described, but we were unable to observe any differences in either early or late apoptosis (Fig. S5A to C). From bead uptake assays, we observed that Dynll1 deficiency also did not appear to affect the phagocytotic capability of DCs (Fig. S6A and B). Together, these results suggested that Dynll1 function is most prominent in intracellular proliferation of *Listeria*, thus indicating that Dynll1 had functions distinct from other autophagy-related proteins.

### Disassociation of Dynll1 from mitochondrial Cox4i1 unleashes intracellular reactive oxygen species.

Dynll1 is known to be a small adaptor-like protein linked to the dynein motor (36), but its relation to immune responses is not fully clear. Recently, Dynll1 has been shown to inhibit the phosphorylation of IκBα to influence NF-κB signaling (37). Therefore, we first explored the possibility that the effects we observed were dependent on IκBα. Western blotting of WT and Dynll1^–/–^ DCs during the first hour of *Listeria* infection showed that Dynll1 deficiency had no effect on the increasing levels of pIκBα present, even though protein expression of Dynll1 also displayed a similar trend in the WT DCs (Fig. S7A and B). Furthermore, DCs were insensitive to treatment with IκBα phosphorylation inhibitor at the start of infection, with significant differences in pathogen burden only seen at very high concentrations (10 μM) of inhibitor (Fig. S7C). Interestingly, Dynll1^–/–^ DCs also displayed increased pathogen burden under this treatment at 6.5 h. These results therefore suggested to us that Dynll1 participation in the signaling pathway of NF-κB may not be an important factor during *Listeria* infection in DC2.4 cells.

**FIG 4:**
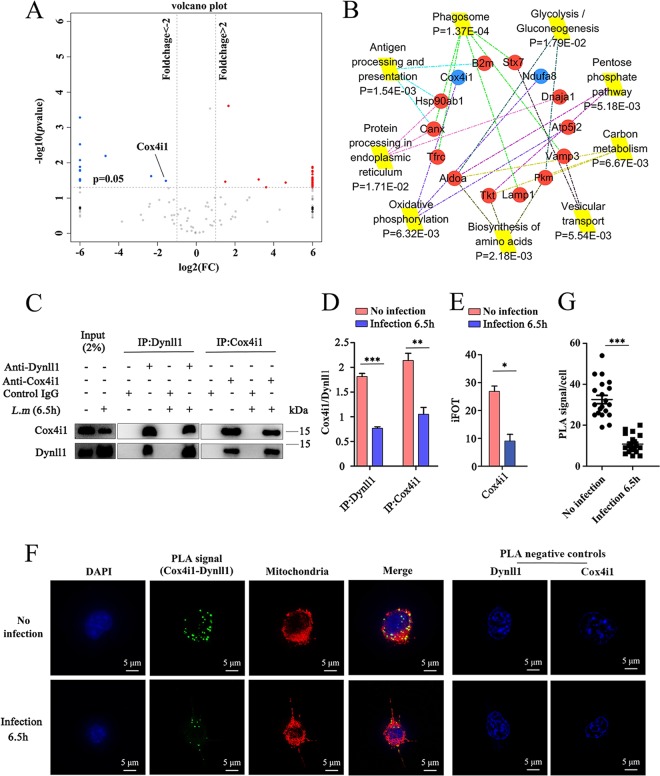
Dynll1 interacting proteins. (A) Differentially expressed proteins in no-infection DCs and DCs infected with L. monocytogenes shown in a volcano plot based on coimmunoprecipitation (Co-IP)-MS analyses. The mean ratios of three biological repeats (infected cells at 6.5 h versus no infection) were plotted on a log_2_ scale (*x* axis) against the corresponding –log_10_ *P* value (*y* axis). Proteins representing fold changes >2 or <0.5 (*P* < 0.05) were considered up- or downregulated and are indicated in red and blue, respectively. (B) KEGG analysis identified major biological pathways in which the differentially expressed proteins were involved. Each colored line indicates a different pathway. KEGG, Kyoto Encyclopedia of Genes and Genomes. Reduction in the levels of the Dynll1-Cox4i1 complex upon *Listeria* infection was shown by Co-IP (C) and quantified (D) (*n* = 3). Co-IP, coimmunoprecipitation. (E) The level of Cox4i1 protein bound to Dynll1 was shown using Co-IP-quantitative MS (*n* = 3). iFOT, the protein abundances. The fraction of total (FOT) was used to evaluate protein abundance, which was calculated as iBAQ of the protein divided by the total iBAQ of all proteins in one sample and then multiplied by 10^5^ for ease of presentation to obtain iFOT. (F) Proximity ligation assays (PLAs) were used to examine interactions between Dynll1 and Cox4i1. Green indicates the PLA signal, and red indicates the mitochondrial dye. (G) Quantification of the PLA signal in no-infection DCs and in DCs infected with L. monocytogenes for 6.5 h. Two-way ANOVA with Tukey-Kramer tests (D) or a two-sided Student *t* test (E and G) was used to measure significance. All data are shown as means ± the SEM. *, *P* < 0.05; **, *P* < 0.01; ***, *P* < 0.001.

**FIG 5:**
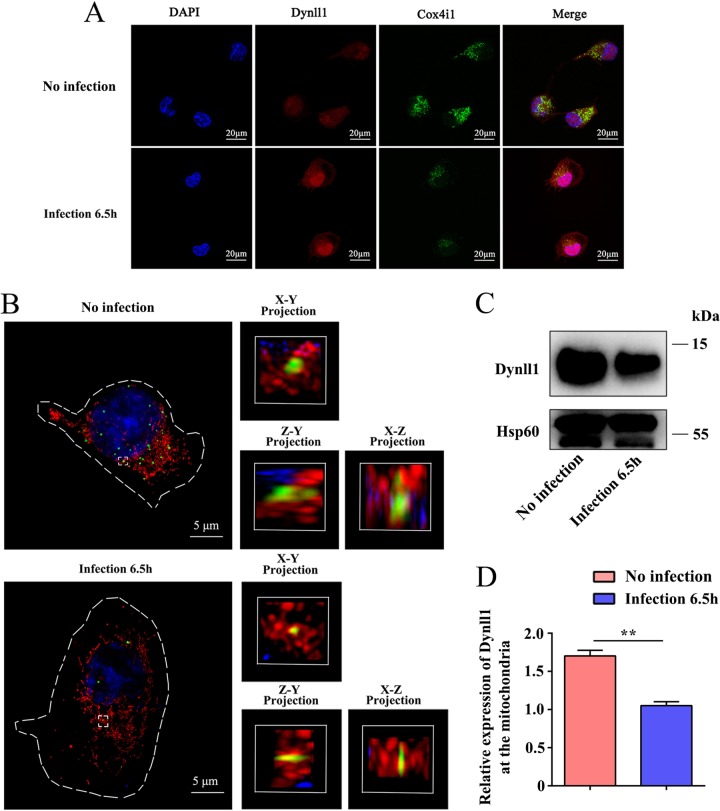
The Dynll1-Cox4i1 complex localizes in the mitochondria. (A) Immunofluorescent staining of Dynll1-Cox4i1 colocalization using confocal microscopy. (B) Localization of the Dynll1-Cox4i1 complex in mitochondria visualized by superresolution microscopy. Red indicates mitochondria, and green indicates the PLA signal (Dynll1-Cox4i1 complex). The level of Dynll1 in mitochondria is displayed (C) and quantified (D) by Western blotting upon *Listeria* infection. A two-sided Student *t* test was used to measure significance. All data are shown as means ± the SEM. *, *P* < 0.05; **, *P* < 0.01; ***, *P* < 0.001.

To gain more insight into the potential role of Dynll1 during *Listeria* infection, we screened for its interacting partners using immunoprecipitation (IP)-MS studies. Western blotting and gel electrophoresis assays were first used to verify the purified Dynll1 protein (Fig. S8A and B). Using IP-MS, we detected 158 possible interacting partners of Dynll1 in DCs (a number that has not been previously reported in existing protein-protein interaction databases; see Table S3 in the supplemental material). Spearman’s correlation analysis of each interacting pair (three replicates for each pair) confirmed that our results were relatively consistent (Fig. S8C), and PCA verified that the coimmunoprecipitated proteins of WT (no infection) and infected DCs were completely separated (Fig. S8D). Differentially expressed proteins between WT (no infection) and infected DCs are shown in a volcano plot (Fig. 4A). Intriguingly, our data predicted that the types of proteins that interacted with Dynll1 were significantly altered by infection (Fig. S9). Therefore, we selected several of the top varied proteins to detect protein interactions using proximity ligation assay (PLA). While we were able to identify protein spots for each complex, Rab1a-Dynll1 (Fig. S10A), Snx2-Dynll1 (Fig. S10B), and Canx-Dynll1 (Fig. S10C) were not measurably altered by infection. In order to further search the signal paths affected by Dynll1, KEGG analysis was employed. The enrichment of proteins involved in oxidative phosphorylation was observed (Fig. 4B). Because Dynll1 itself is not known to be an MP and our results showed that it may have a role distinct from traditional autophagy, we investigated whether Dynll1 interacted with any known MPs involved in oxidative phosphorylation. We found that the interaction between Dynll1 and the mitochondrial respiratory chain member Cox4i1 was significantly lowered after infection (Fig. 4A). Importantly, Cox4i1 was detected in the IP-MS of the WT and infected DCs, unlike other proteins that were detected under only one condition, such as Rab1a, Stx7, B2m, and Vamp3 (Table S3). Forward and reverse coimmunoprecipitation (Co-IP) assays confirmed that Dynll1 and Cox4i1 could interact and that the extent of the interaction was reduced by infection (Fig. 4C and D; see also Fig. S11). These results were also consistent with our Co-IP-MS data, which showed decreased levels of Cox4i1 bound to Dynll1 in infected DCs compared to WT cells (Fig. 4E). PLA showed that the interaction spots of the complexes were concentrated on mitochondria and were not cytoplasmic (Fig. 4F), and the authenticity of the PLA signal was confirmed by negative control. Quantification of the PLA signals indicated decreasing levels of the Dynll1-Cox4i1 complex (Fig. 4G). Confocal microscopy further confirmed these results and showed that the colocalization of Dynll1 and Cox4i1 was primarily determined by Cox4i1 distribution (Fig. 5A). In addition, ultra-high-resolution microscopy showed that the protein complex was located inside the mitochondria (Fig. 5B). In order to further verify the loss of Dynll1 from mitochondria following infection, we extracted mitochondria for Western blot analysis, and the results showed that the extent of Dynll1 was significantly reduced by infection (Fig. 5C and D and S12).

**FIG 6:**
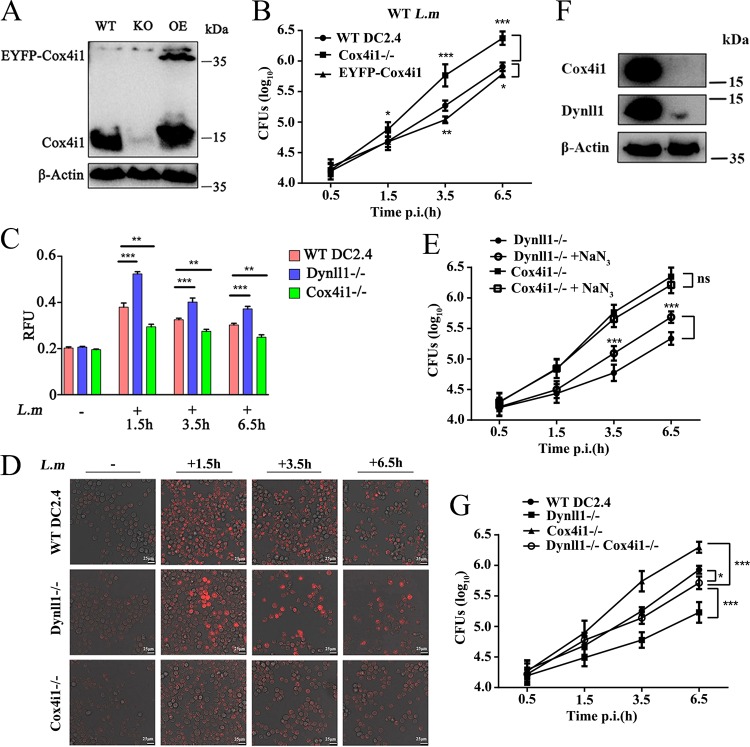
Dynll1 disassociation from mitochondrial Cox4i1 unleashes mitochondrial reactive oxygen species. (A) Verification of Cox4i1 knockout and EYFP-Cox4i1-overexpressing DCs by Western blotting. (B) CFU of L. monocytogenes in WT, Cox4i1 KO, and EYFP-Cox4i1 cells after infection (*n* = 8). KO, knockout; EYFP, enhanced yellow fluorescent protein. Quantification (C) (*n* = 3) and visualization (D) of the levels of mitochondrial reactive oxygen species in WT, Cox4i1, and Dynll1 KO DCs at different time points after L. monocytogenes infection using MitoSOX red mitochondrial superoxide indicator. (E) L. monocytogenes amounts in Dynll1 and Cox4i1 KO DCs incubated with 5 nM NaN_3_ after infection (*n* = 8). (F) Verification of Dynll1 and Cox4i1 double-KO DCs by Western blotting. (G) CFU of L. monocytogenes in WT, Dynll1 KO, Cox4i1 KO, and Dynll1-Cox4i1 double-KO DCs after infection (*n* = 8). Two-way ANOVA and Tukey-Kramer tests were used to measure significance. All data are shown as means ± the SEM. *, *P* < 0.05; **, *P* < 0.01; ***, *P* < 0.001.

To test whether Dynll1 exercised its functions through interaction with Cox4i1, we generated Cox4i1 knockout and overexpression DC2.4 cell lines (Fig. 6A) and assessed the functional significance of Cox4i1 during infection. As expected, Cox4i1 deficiency led to a clear increase in intracellular *Listeria* (Fig. 6B). Moreover, the overexpression of Cox4i1 in WT DCs showed a slight but significant decrease in bacterial viability compared to control WT DCs. At the same time, Cox4i1^–/–^ depletion had no effect on cell apoptosis during *Listeria* infection within 6.5 h of infection (Fig. S13A to C).

**FIG 7:**
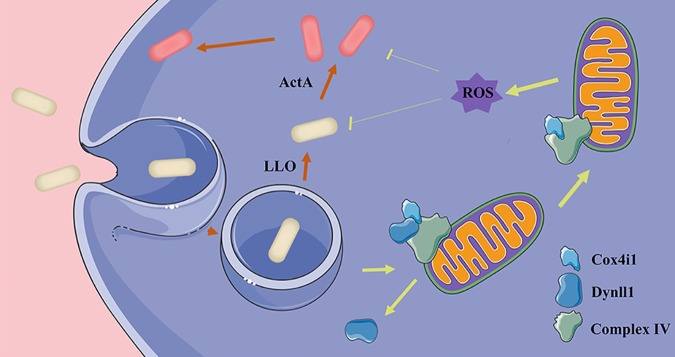
Illustration of Dynll1-Cox4i1 complex response to intracellular bacterial attack. Dynll1 binding with Cox4i1 inhibits the release of reactive oxygen species in resting cells. When the host cell senses *Listeria* invasion, the Dynll1-Cox4i1 complex of mitochondria dissociates to release reactive oxygen species to kill intracellular *Listeria*.

Because Cox4i1 is known to play an important role in regulating mitochondrial oxygen production (38), we analyzed the relative content of mitochondrial reactive oxygen species (ROS) during the course of infection using mitochondrial ROS indicator using a dye specific for superoxide. Although the ROS levels significantly spiked at 1.5 h postinfection and persisted throughout the 6.5-h observation window in WT cells, the oxidative burst was more subdued in Cox4i1^–/–^ cells (Fig. 6C and D). However, depletion of Dynll1 significantly increased the strength and duration of the mitochondrial oxidative burst (Fig. 6C and D). In addition, the results showed that the addition of sodium azide (the use of the general cytochrome *c* oxidase inhibitor) significantly increased the CFU of L. monocytogenes in Dynll1-deficient cells but had no effect on Cox4i1-deficient cells, further demonstrating the importance of the mitochondrial oxidative burst on bacterial viability (Fig. 6E). In order to clarify that the bacterial inhibition observed was indeed dependent on the protein complex, we constructed Dynll1^–/–^ and Cox4i1^–/–^ double-knockout cells (Fig. 6F). Interestingly, the *Listeria* burden in these cells following infection was much closer to the levels in WT cells, albeit still significantly lower (Fig. 6G). This result suggests that the function of Cox4i1 explained a major part of the reduction in *Listeria* seen following Dynll1 depletion. Collectively, our data demonstrate that the respiratory chain complex factor Cox4i1 also plays an important role in host defense against *Listeria* by triggering mitochondrial ROS in response to Dynll1 dissociation (Fig. 7).

## DISCUSSION

Changes in mitochondrial dynamics have long been known to play important roles in host responses to intracellular pathogens (39). Previous studies on *Listeria* and other infections have shown that significant changes in mitochondrial distribution and membrane potential can occur shortly after exposure to insult via PRR signaling (39–41). Damaged mitochondria by themselves may also serve to propagate inflammatory responses in the cell by serving as lipid antigens and as a source for free nucleic acids (42, 43). Macrophages are known to use mitochondria to generate ROS to kill antigens captured in lysosomes and even potentially burst mitochondrial DNA to serve as a scaffold for extracellular traps for pathogens (44, 45). However, the role of the mitochondria after pathogen escape from the lysosome remains unclear. In this report, we observed that the respiratory chain complex member Cox4i1 played a role in the killing of intracellular *Listeria* by promoting the release of mitochondrial ROS. This release appears to be far less intense than the oxidative bursts that occur in activating neutrophils (46); however, it is likely to have broad effects considering the potency of ROS signaling pathways. Intracellular ROS are known to alter the functions of a range of different cellular proteins (47), including a number of the MPs, such as Mst and Nrf2 (48), and different ROS species may mediate distinct effects. How these different ROS species may trigger host responses or otherwise directly disrupt bacterial processes critical for the intracellular survival of *Listeria* remains to be explored.

From our data, it appears that the Cox4i1-triggered mitochondrial ROS signals may not originate from the direct contact of *Listeria* with the mitochondrion after lysosomal escape; instead, it is triggered by the disassociation of Dynll1 from Cox4i1. Dynll1 is a subunit of the dynein motor complex and is known for its role in vesicular transport (36). However, more recent work has shown that it may participate in other processes, such as nuclear factor κB (NF-κB) activation, development, and expansion of MYC-driven lymphomas and innate B cell development (36, 37, 49–52). These additional roles may require Dynll1 to interact with additional partners in different organelles. Although we found Dynll1 to be involved in a repressive complex with Cox4i1, we also identified more than 100 other proteins by IP-MS analyses, some of which may be potential interacting partners of Dynll1. The Dynll1-Cox4i1 complex itself also may have other mechanisms to combat *Listeria* infection, as reflected in our data. Further exploration of the biological role of Dynll1-Cox4i1 complex may help us understand the molecular functional diversity in DCs. Further exploration of these interactions may shed light on the mechanisms by which Dynll1 performs a variety of cellular functions.

The Dynll1-Cox4i1 complex may only be a part of a complex web of protein interactions that are involved in the defense response of DCs to intracellular pathogens. From our membrane proteomics data, a large number of other differentially expressed MPs were found and may also play important roles in ensuring that DCs successfully balance infection prevention with efficient presentation of antigens to other immune cells. Future integration of our membrane proteomics data with metabolomics and transcriptome data sets may help clarify the overall landscape of DC responses to intracellular bacterial pathogens.

However, the swift yet relatively mild response mediated by the Dynll1-Cox4i1 complex in response to bacterial escape from the lysosome suggests that this complex may be one of the early signaling events that occur in DCs upon initial detection of intracellular pathogen escape. This mechanism is particularly interesting since the bacterial killing benefit provided would likely come at the expense of reduced mitochondrial respiratory capacity. Additional research into the timing and relative intensities of DC defense responses may help clarify whether DCs truly operate via progressively intensifying signals to manage intracellular pathogens. An understanding of this system may have significant ramifications in guiding design of antibiotics against intracellular pathogens to operate under novel mechanisms.

## MATERIALS AND METHODS

### Cells.

The murine bone marrow-derived dendritic cell line (DC2.4) was kindly provided by K. Rock (Dana Farber Cancer Institute, Boston, MA) as previously described (17, 53). DC2.4 cells were cultured in Roswell Park Memorial Institute (RPMI) 1640 medium containing 2 mM l-glutamine and 10% heat-inactivated fetal bovine serum. Cells were incubated at 37°C in 5% CO_2_.

### Bacterial strain culture and infection.

L. monocytogenes strains were stored at –80°C in brain heart infusion (BHI) medium containing 50% glycerol and cultured in BHI medium (BD Biosciences). Selected colonies were grown overnight at 30°C with shaking. Bacteria were pelleted, washed, and resuspended in phosphate-buffered saline (PBS). The bacterial inocula were estimated based on the optical density at 600 nm (OD_600_) and verified by plating serial dilutions on plates to determine the CFU.

L. monocytogenes WT (10403S), Δ*hly* (DP-L2161, ΔLLO), and Δ*actA* (DP-L1942, ΔActin) strains, as well as the L. monocytogenes RFP (DP-L5538) strain in which the RFP tag is expressed under the *actA* promoter, were saved in our laboratory (17). DCs of 8 × 10^4^ cells/well were plated into 96-well plates (eight or four independent wells according to experiment needs) and cultured for 8 h. DCs were infected with L. monocytogenes strains, which were cultivated in BHI medium (BD Biosciences) to an OD_600_ of 0.4 at a multiplicity of infection (MOI) of 20. At 1 h postinfection, the culture medium was replaced with 100 μg/ml gentamicin for 30 min to kill extracellular bacteria, followed by treatment with 10 μg/ml gentamicin to prevent reinfection. At each time point, DCs were lysed with 0.1% Triton (Sigma) for 10 min and then serially diluted and plated on BHI plates (the samples were tested in triplicate) to verify the colony count.

### Membrane protein purification for MS analysis.

DC2.4 dendritic cells were harvested and washed three times with PBS. Membrane proteins were extracted using a Minute Plasma membrane protein isolation and cell fractionation kit (Invent Biotechnologies, SM-005) according to the manufacturer’s instructions. Protein concentration was determined using a Bradford protein assay kit (P0006; Beyotime, China). Samples (200 μg each) containing extracted membrane proteins were digested with trypsin, as described previously (54). Tryptic peptides were preseparated on a Waters 2695 HPLC system equipped with a C_18_ column (5 μm, 4.6 × 250 mm; Thermo Fisher) and eluted using a continuous acetonitrile gradient as follows: time = 0 min, 100% A (water), 0% B (acetonitrile), 0.5 ml/min; time = 35 min, 2% A (water), 98% B (acetonitrile), 0.5 ml/min; and time = 40 min, 2% A (water), 98% B (acetonitrile), 0.5 ml/min. The peptides were carefully collected into 40 microcentrifuge tubes based on the eluting time (1 min/tube) and combined to form eight fractions. The peptides were then dried in a vacuum concentrator and dissolved in 0.1% formic acid. LTQ Orbitrap Velos Pro equipped with an Easy-nLC 2000 Nanoflow high-performance liquid chromatography system (3 μm, 0.075 × 150 mm; Thermo Fisher) was used for MS analyses. Peptides were separated in the C_18_ column with the following gradient: time = 0 min, 98% A (0.1% formic acid in water), 2% B (0.1% formic acid in acetonitrile); time = 5 min, 95% A, 5% B; time = 90 min, 65% A, 35% B; time = 110 min, 2% A, 98% B; and time = 120 min, 98% A, 2% B, at a flow rate of 200 nl/min. The MS and MS^n^ (multistage mass spectrometry) analysis conditions were the same as those we described previously (17).

### Protein identification and quantification.

The raw data were searched against a mouse Refseq protein database by using Mascot implemented on Proteome Discoverer 1.4 (PD1.4). The mass tolerances were set at 20 ppm for precursors and 0.5 Da for product ions. The cysteine carbamidomethylation as a fixed modification and N-terminal acetylation and oxidation of methionine as variable modifications were applied in PD1.4. One missed cleavage was accepted, and the FDR of peptide levels was controlled at 1%. An intensity-based absolute quantification (iBAQ) approach was used for protein quantification. Briefly, the fraction of total (FOT) was used to evaluate protein abundance, which was calculated as the iBAQ of the protein divided by the total iBAQ of all proteins in one sample and then multiplied by 10^5^ for ease of presentation to obtain the iFOT.

### Proteome data filtering and bioinformatics analysis.

The proteins which were detected in at least 3 of 6 experiments were picked for bioinformatics analysis. The differentially expressed proteins that showed a >2-fold change between paired samples (*P* < 0.05 [by using paired two-tailed student test]) were selected. The WebGestalt system (http://www.webgestalt.org/) was used for GO enrichment and KEGG analysis, and the significance level was set at *P* < 0.05, using all identified proteins as reference. The STRING database (https://string-db.org/) was used for network analysis, and the interaction score was set to high confidence (scores > 0.7). Protein-protein interaction networks were displayed using Cytoscape software.

### Establishment of knockout, knockdown, and overexpression DCs.

The 3GGGGS-Cox4i1 sequence was synthesized by Beijing Genomics Institute (BGI, China), and then the sequence was linked between the BsrG1 and EcoR1 sites of FUGW-EYFP plasmid by T4 DNA ligase. sgRNAs of Dynll1, Cox4i1, Atg2b, and Atg4b were also synthesized according to the following sequences: Dynll1 forward (5′-CACCGGAGAAGTACAACATCGAGA-3′) and reverse (5′-AAACTCTCGATGTTGTACTTCTCC-3′); Cox4i1 forward (5′-CACCGTCACGCCGATCAGCGTAAG-3′) and reverse (5′-AAACCTTACGCTGATCGGCGTGAC-3′); Atg2b forward (5′-CACCGATCCAGGCTCAGCTGCTCC-3′) and reverse (5′-AAACGGAGCAGCTGAGCCTGGATC-3′); and Atg4b forward (5′-CACCGAGAGTATCATATGTCAAAG-3′) and reverse (5′-AAACCTTTGACATATGATACTCTC-3′). They were then linked in the U6-sgRNA-SFFV-spCas9-puro plasmid by T4 ligase between two BbsI sites. Knockout and overexpression DCs were established by infection of high-titer lentiviral particles in our laboratory. Briefly, 293T cells were cotransfected with 1 μg of pMD2.G, 1.5 μg of psPAX2, and 2 μg of Cas9 plasmid or 2 μg of overexpression plasmid in 2-cm plates mixed with 15 μl of Lipofectamine 2000 (Invitrogen) when the cell confluence reached about 80%. Media were refreshed after incubation for 8 h, and supernatants containing virus particles were collected by centrifugation after 48 h of cultivation. DCs were infected with virus particles at an MOI of 100. Overexpressed positive cells were collected by using a FACSAria II SORP (BD Bioscience) and verified by Western blotting. Knockout and knockdown DCs were cultured in 0.5 μg/ml puromycin for 3 days to select positive cells and then were seeded into 96-well plates via single cell sorting. Western blotting was used to screen knockout- and knockdown-positive cells following the establishment of these cell lines.

### Immunofluorescence assays.

Cells were washed three times with PBS, fixed with 4% paraformaldehyde for 10 min, and then permeabilized in 0.2% Triton X-100 for 10 min. QuickBlock blocking buffer (P0252; Beyotime) was used to block nonspecific antigens for 2 h. Subsequently, the cells were incubated overnight with primary antibodies at 4°C. The cells were then incubated with secondary antibodies conjugated with Alexa Fluor 488 (Cell Signaling Technology [CST], 4412) or Alexa Fluor 594 (CST, 8889) for 1 h at 37°C. Finally, the cells were stained with DAPI (4′,6′-diamidino-2-phenylindole, catalog no. D9542; Sigma-Aldrich) for 10 min, washed three times with PBS, and analyzed by confocal microscopy with a Leica SP8 fluorescence microscope.

### Superresolution microscopy.

A DeltaVision OMX V4 Blaze (GE Healthcare) was used for 3D-structured illumination by superresolution microscopy. Briefly, pixel registration was corrected to be <1 pixel for all channels using 100-nm Tetraspeck beads (Molecular Probes). SoftWoRx v.6.0 software was used to reconstruct superresolution 3D images.

### Immunoblot assays.

Membrane protein, cytoplasm protein, and whole protein were extracted using a Minute Plasma membrane protein isolation and cell fractionation kit. Portions (20 μg) of protein were resolved by sodium dodecyl sulfate-polyacrylamide gel electrophoresis (SDS-PAGE; Beyotime) and blotted on polyvinylidene difluoride membranes (Invitrogen). The proteins were then probed with Dynll1 (Abcam), Cox4i1 (CST), ATG4B (CST), mTOR (CST), β-Actin (Thermo Fisher), ATG2B (Abcam), IκBα (CST), p-IκBα (CST), or vinculin (CST) primary antibodies, followed by horseradish peroxidase-conjugated anti-rabbit (CST) or anti-mouse (CST) secondary antibodies. Signals were detected using the SignalFire Plus ECL reagent (CST).

### Coimmunoprecipitation assays.

A total of 1 × 10^8^ DCs were collected and lysed using radioimmunoprecipitation assay buffer. DC supernatants were added to 50% preprocessed protein A-agarose (CST) at a ratio of 100 μl per 1 ml of sample solution. The supernatant solutions were shaken on a horizontal shaker for 90 min at 4°C. They were then transferred to new tubes and mixed with appropriate amounts of primary antibodies, according to manufacturer’s specifications, and shaken overnight on a horizontal shaker at 4°C. The preprocessed protein A-agarose was then added to the supernatant solutions, and the tubes were placed on a rotating shaker at 16°C for 2 h. The pellets were washed with cold PBS three times and then used for SDS-PAGE, Western blot, and HPLC-MS analyses. The MS data were obtained via subtracting the proteins detected by IgG control MS from the all proteins detected by Dynll1 IP-MS.

### Cytophagy assays.

Cytophagy assays of DCs were performed using a FluoSpheres size kit 2 (Thermo Fisher, F8888) according to the manufacturer’s instructions. Briefly, DCs were placed into six-well plates at 1 × 10^5^ cells/well and cultured for 12 h. Then, the DCs were infected with FluoSpheres beads at an MOI of 20 for 1 h. The DCs were collected and washed with PBS for flow cytometry detection.

### Reactive oxygen species detection.

ROS detection was performed using MitoSOX red mitochondrial superoxide indicator (Thermo Fisher, M36008). Briefly, DCs were covered in 1 ml of 5 μM MitoSOX reagent working solution, incubated for 10 min at 37°C, and gently washed three times with warm PBS. Immediately, the DCs were analyzed by confocal microscopy using the Leica SP8 fluorescence microscope and a Varioskan LUX reader.

### Duolink proximity ligation assay.

A Duolink PLA was performed as described previously (Sigma) (17). Briefly, 1 × 10^4^ cells were plated on a glass slide, fixed, and blocked using the Duolink blocking solution (Sigma). The cells were coincubated overnight with anti-Dynll1 (Abcam) and mouse-Cox4i1 (CST) antibodies at 4°C. The secondary antibodies of the Duolink PLA probe were added to the glass slide; the ligation mixture was then added to the surface of the glass slide. The signal was amplified and imaged with green-labeled oligonucleotide detection probes using a DeltaVision OMX V4 Blaze and a Leica SP8 fluorescence microscope. The implementation of negative controls of PLA was required. The main purpose was to verify the specificity of the antibody so that the images had no additional background. Briefly, only one antibody (Dynll1 or Cox4i1) was added in a glass slide, and the glass slide was incubated overnight at 4°C. Other procedures referred to the Duolink PLA.

### Quantification and statistical analysis.

Data were statistically analyzed using GraphPad Prism 5. Where indicated, the mean of at least three independent experiments is presented, with error bars showing standard deviation (SD) or the standard error of the mean (SEM), as indicated in the figure legends. Immunofluorescent images or the intensity of the Western gel was analyzed using ImageJ. Two-way analysis of variance (ANOVA), along with Tukey-Kramer tests or two-sided Student *t* tests, was used to measure significance as described in the figure legends. A *P* value of <0.05 was considered significant (*, *P* < 0.05; **, *P* < 0.01; ***, *P* < 0.001).

### Data availability.

The MS proteomics data were deposited in the ProteomeXchange Consortium under data set identifier PXD016452.

## Supplementary Material

Supplemental file 1

Supplemental file 2

Supplemental file 3

Supplemental file 4
