# Supplementary material for: The Dynll1-Cox4i1 Complex Regulates Intracellular Pathogen Clearance via Release of Mitochondrial Reactive Oxygen Species
Source: Infect Immun. 2020 Mar 23;88(4):e00738-19. doi: 10.1128/IAI.00738-19 (PMC7093135; doi:10.1128/IAI.00738-19)
Supplement: Supplemental file 1 [file IAI.00738-19-s0001.pdf]

Supplementary Materials:

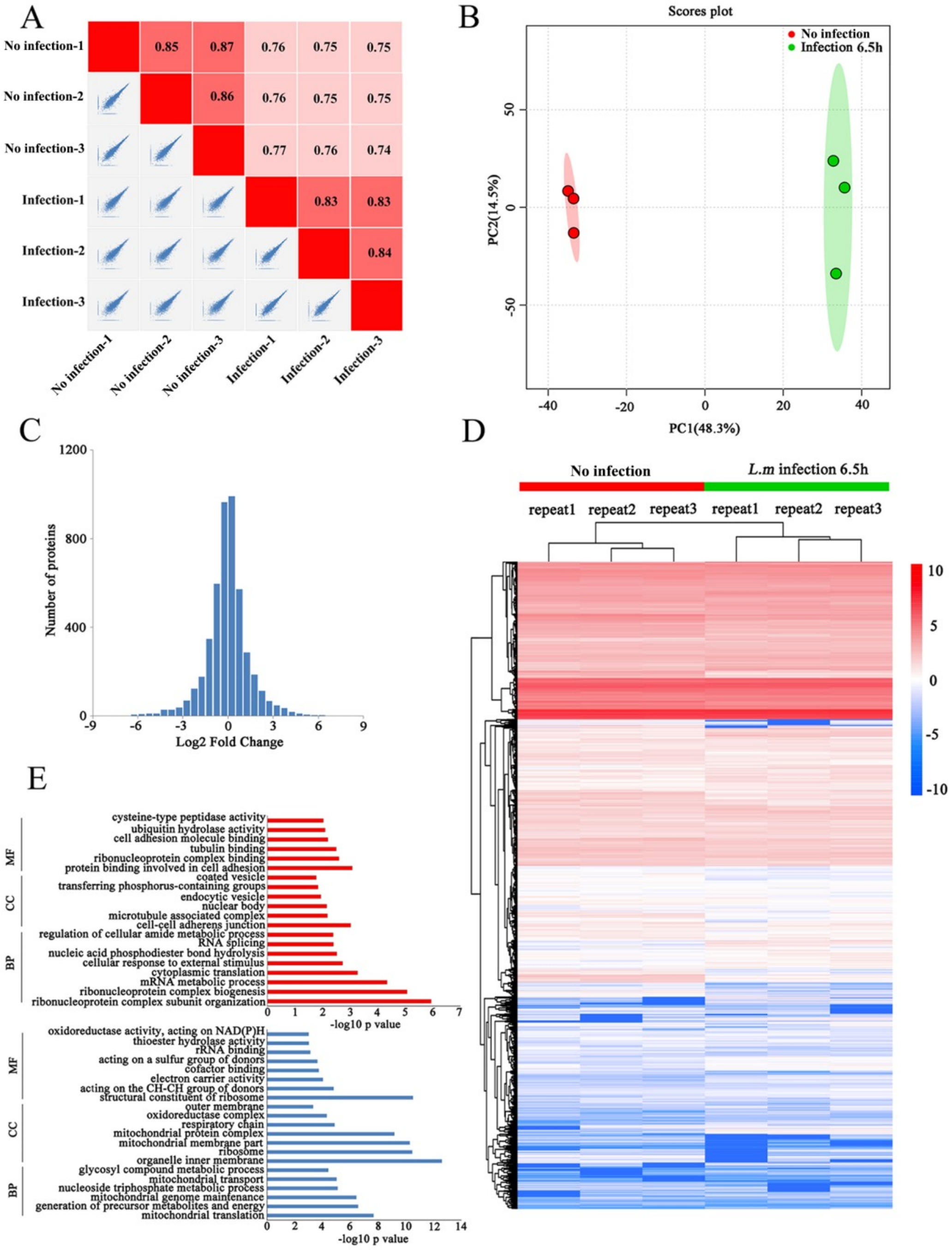

Figure S1. Quality control and bioinformatics analysis using label-free membrane protein quantitative mass spectrometry

(A) Spearman’s correlation coefficients were calculated to assess the reproducibility

experiments. (B) Principal component analysis (PCA) was performed to distinguish No infection and dendritic cells infected with *L. monocytogenes*. (C) Histogram depicting the distribution of protein-abundance ratios. The fold changes of proteins in dendritic cells infected with *L. monocytogenes* versus No infection are shown on a log2 scale on the x-axis and the y-axis shows the number of proteins. (D) Heat-map visualization of 4783 proteins identified using label-free quantitative mass spectrometry. Changes in protein expression are depicted by a range of red and blue intensities (n = 3). (E) Gene ontology enrichment analysis of up-regulated proteins (red bar) and down-regulated proteins (blue bar) involved in molecular function, cell component, and biological process.

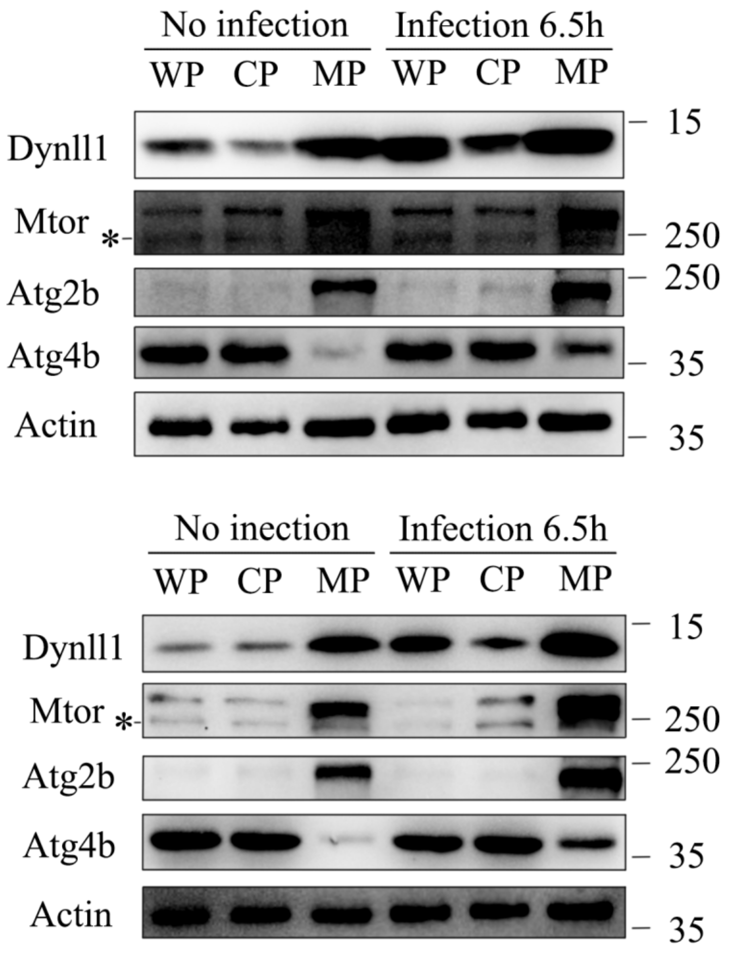

Figure S2. Western blot detect four autophagy-related proteins in No infection and infected dendritric cells

The independent WB experiments are used to generate the quantitative data for Fugure2E. WP: whole protein; CP: cytoplasm protein; MP: membrane protein.

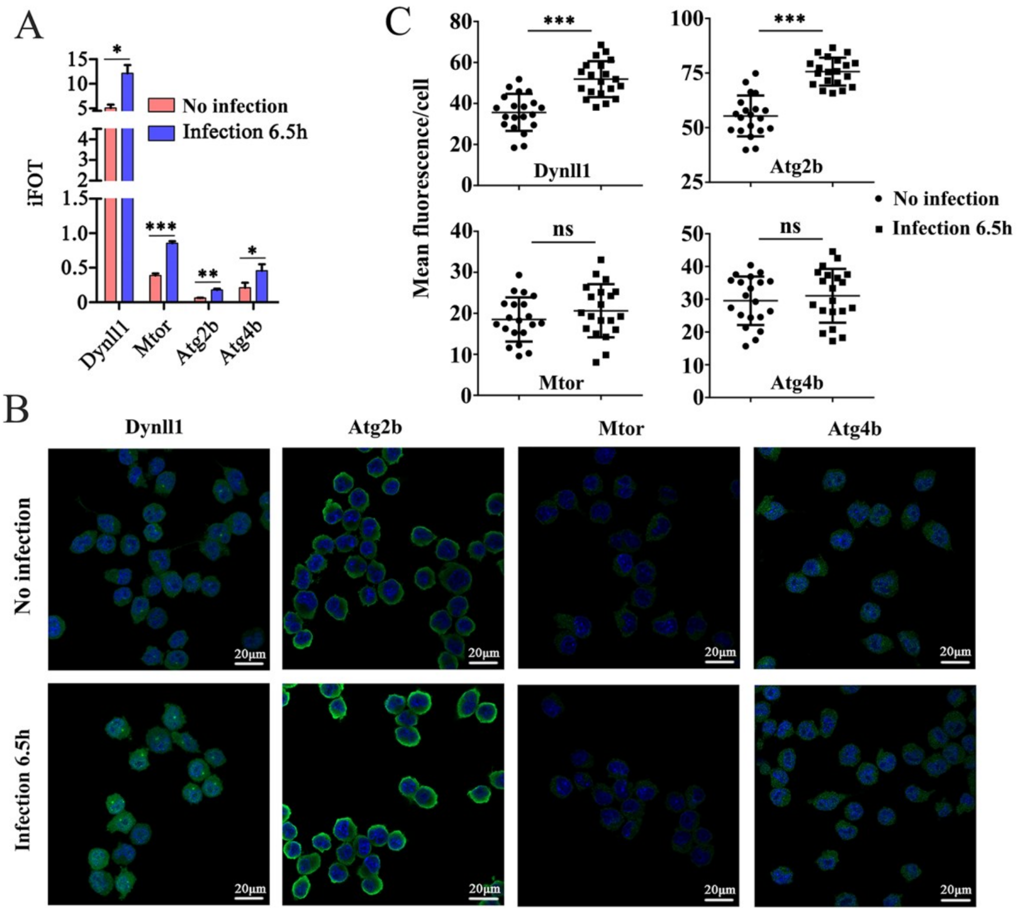

Figure S3. Biological validation of differentially expressed proteins in autophagy-related modules and pathways as a result of *L. monocytogenes* infection

(A) Four autophagy-associated components which were differentially expressed in infected cells at 6.5 h versus No infection cells were measured by mass spectrometry (n = 3). iFOT, the expression profile of membrane peoteins. The fraction of total (FOT) was used to evaluate protein abundance, which was calculated as iBAQ of the protein divided by the total iBAQ of all proteins in one sample and then multiplied by 10^5 for ease of presentation to obtain iFOT. Two-way ANOVA with Tukey-Kramer tests were used to measure significance. Visualization (B) and quantification (C) of Dynll1,

mTor, Atg2b, and Atg4b expression by immunofluorescence in No infection and dendritic cells infected with *L. monocytogenes* for 6.5 h. Two-sided Student's t test was used to measure significance. All data are shown as mean ± SEM. * $p < 0.05$, ** $p < 0.01$ and *** $p < 0.001$.

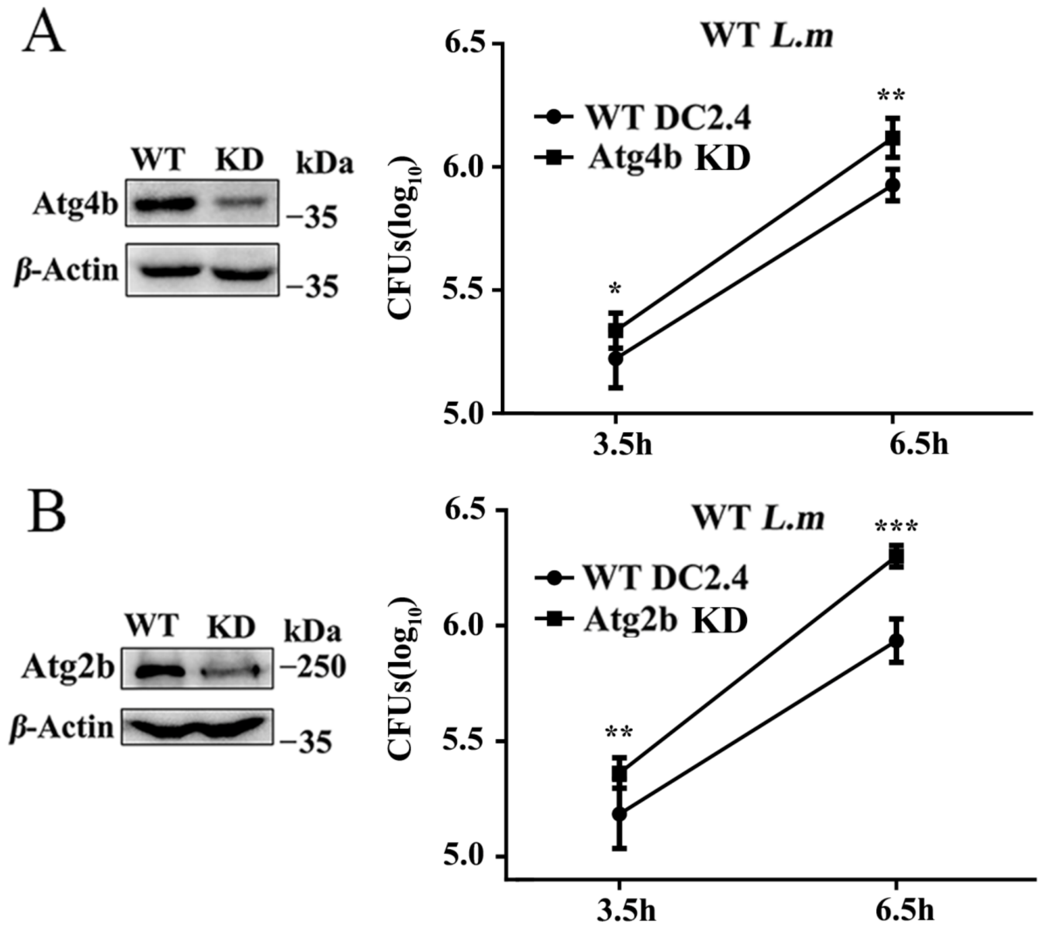

Figure S4. Atg4b and Atg2B control intracellular *L. monocytogenes* growth in dendritic cells

Colony-forming units (CFUs) recovered from WT and Atg4b knockdown cells (A); WT and Atg2b knockdown cells (B), after infection with *L. monocytogenes* for 3.5 and 6.5 h (n = 8). KD, knockdown; DC, dendritic cell. Two-way ANOVA with Tukey-Kramer tests were used. All data are shown as mean ± SEM. * $p < 0.05$, ** $p < 0.01$ and *** $p < 0.001$.

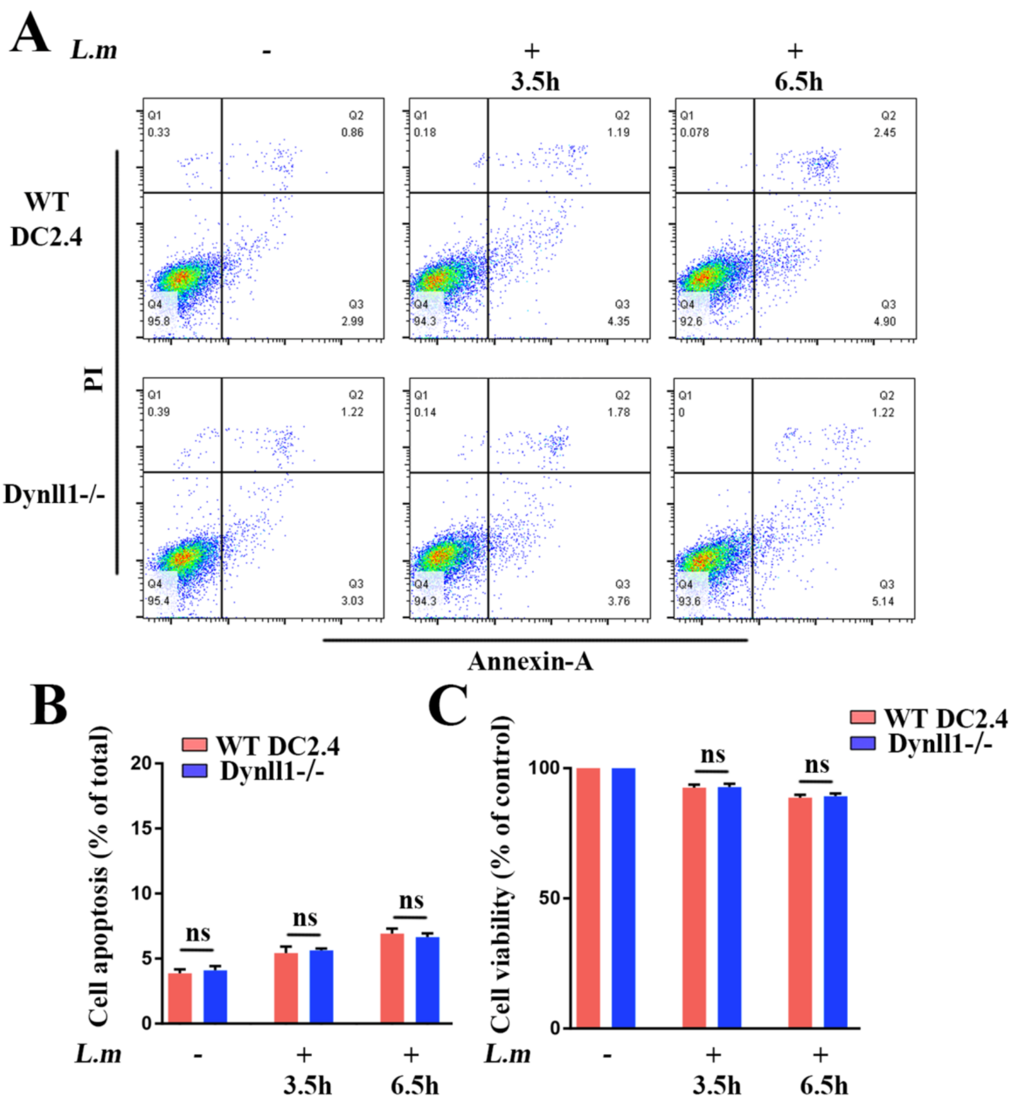

Figure S5 Deletion of Dynll1 has no effect on host cell death upon *L. monocytogenes* infection

Representative flow cytometric plots (A) and statistical analysis (B) depict apoptosis of WT and Dynll1-/- mutant DCs upon *L. monocytogenes* infection for 3.5h and 6.5h (n=3). (C) Quantification of living cells of WT and Dynll1-/- mutant DCs use Cell Counting Kit-8 during *L. monocytogenes* infection for 3.5h and 6.5h (n=3). Two-way ANOVA with Tukey-Kramer tests were used to measure significance. All data are shown as mean ± SEM. * $p < 0.05$, ** $p < 0.01$ and *** $p < 0.001$.

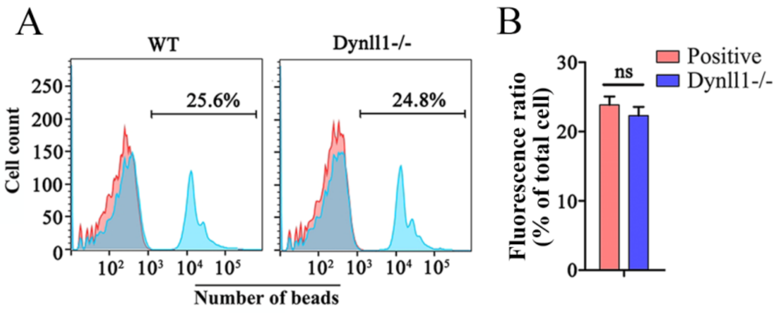

Figure S6 Deletion of Dynll1 does not affect the phagocytosis of host cells

(A and B) The ratio of WT and Dynll1 knockout dendritic cells phagocytosing fluorescent microspheres is shown (n = 3). Two-sided Student's t test was used to measure significance. All data are shown as mean ± SEM. * $p < 0.05$, ** $p < 0.01$ and *** $p < 0.001$.

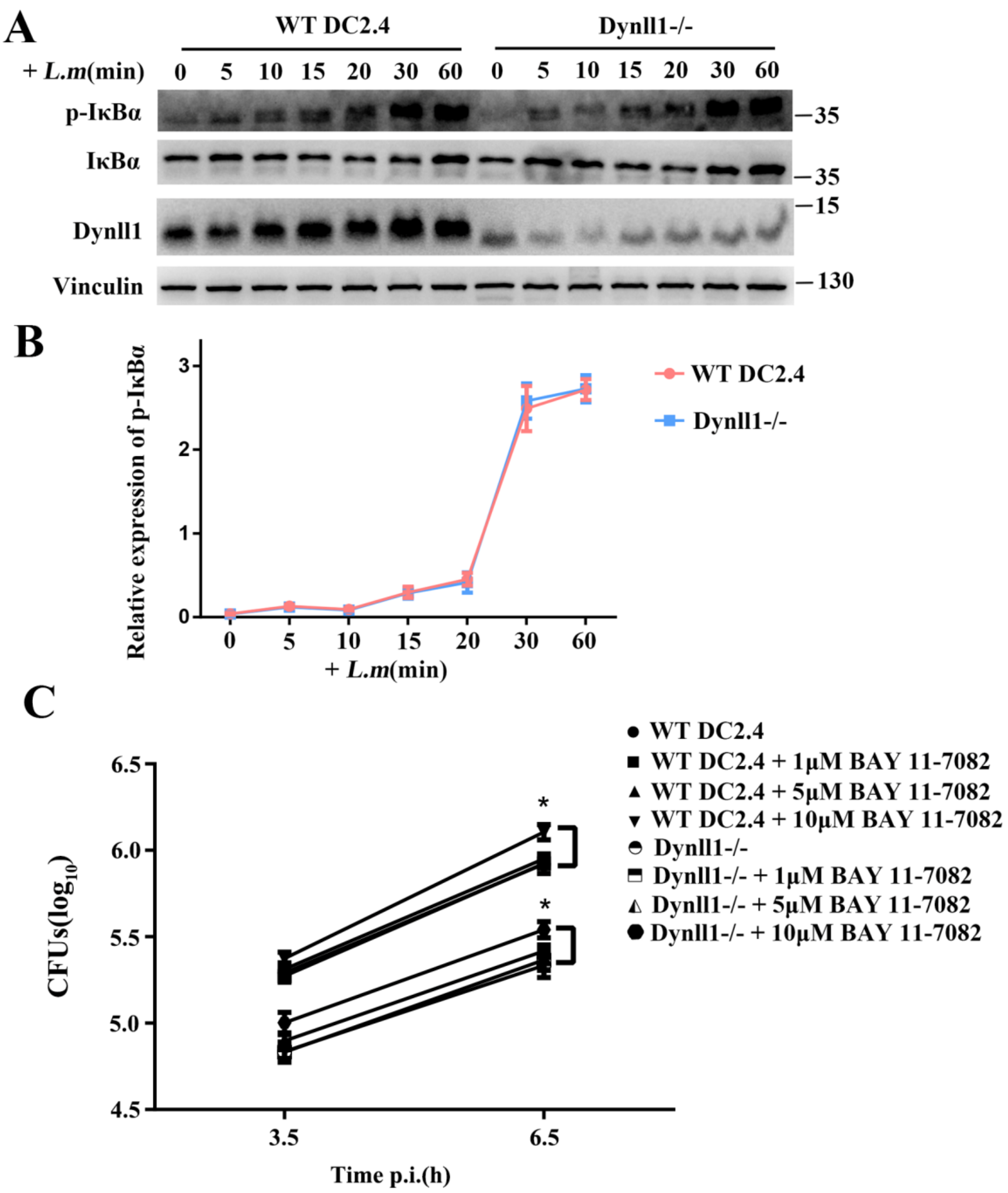

Figure S7 Dynll1 is not involved in NF-κB signal activation in DCs during *L. monocytogenes* infection

Immunoblot analysis of p-IκBα (A) and normalized by vinculin (B) during *L. monocytogenes* infection for the indicated times (n=3). (C) Intracellular *L. monocytogenes* amounts in WT, and Dynll1 KO dendritic cells incubated with NF-κB signaling pathway inhibitors (BAY 11-7082) of different concentrations (1μm, 5μm and 10μm) after infection for 3.5 and 6.5 h (n = 4). Two-way ANOVA with

Tukey-Kramer tests were used to measure significance. All data are shown as mean ± SEM. * $p < 0.05$

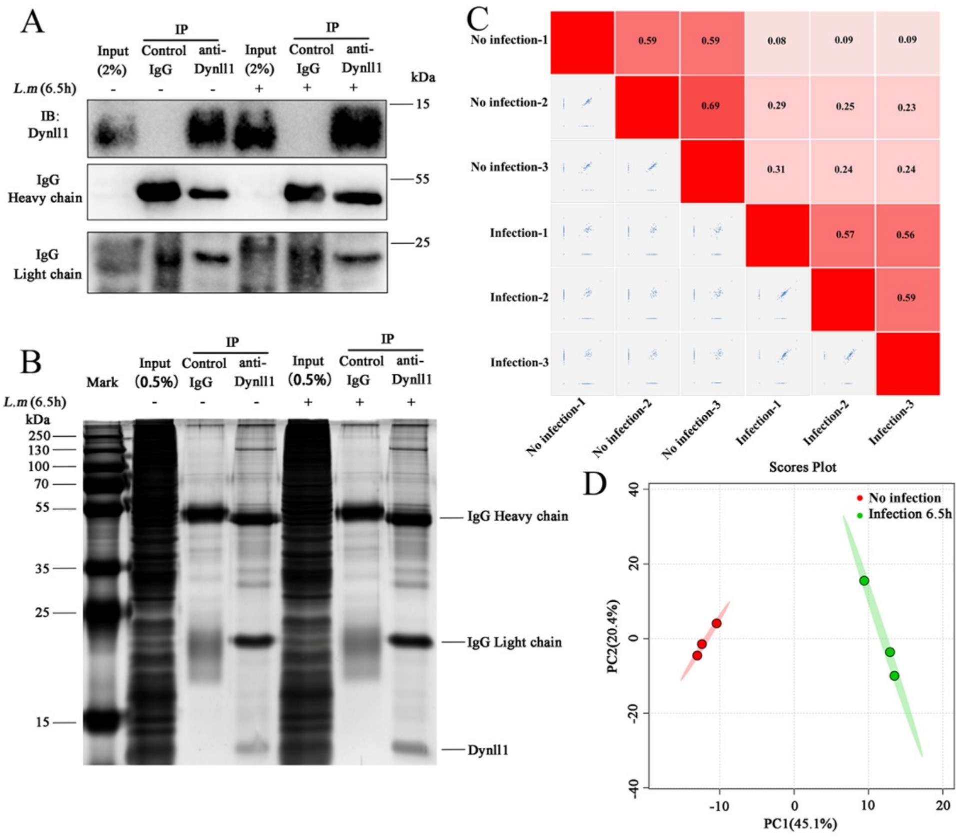

Figure S8. Quality control of Dynll1 for Co-IP mass spectrometry

Western blotting (A) and Coomassie Brilliant Blue staining after gel electrophoresis (B) were used to detect Dynll1 co-immunoprecipitants for pre-mass spectrometry. (C) Spearman's correlation coefficients were calculated to assess the reproducibility of experiments. (D) PCA was performed to distinguish Co-IP samples of No infection from those of infected dendritic cells.

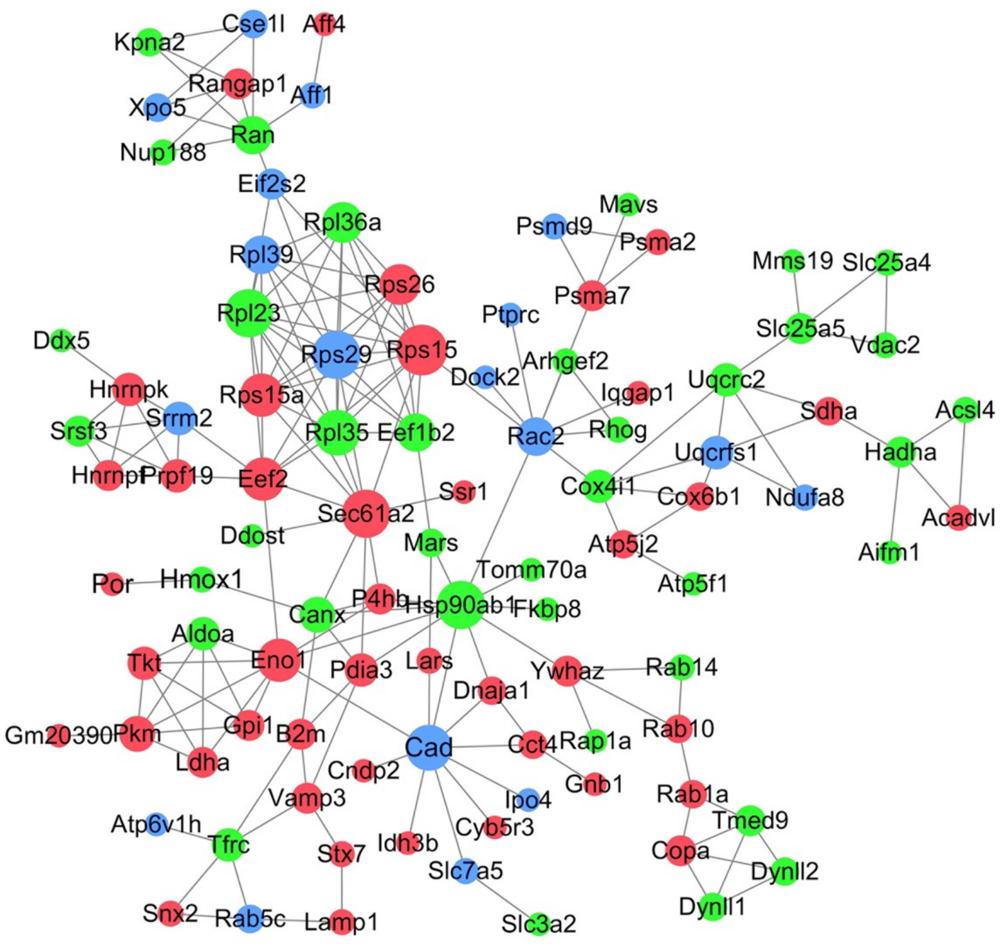

Figure S9. Network analysis of proteins identified by Co-IP quantitative mass spectrometry

Blue was unique to proteins of No infection; red was unique to proteins of DC2.4 infected with *L. monocytogenes* and green represented proteins shared by both samples. Sizes of nodes were indicated based on the numbers of interacting neighbors.

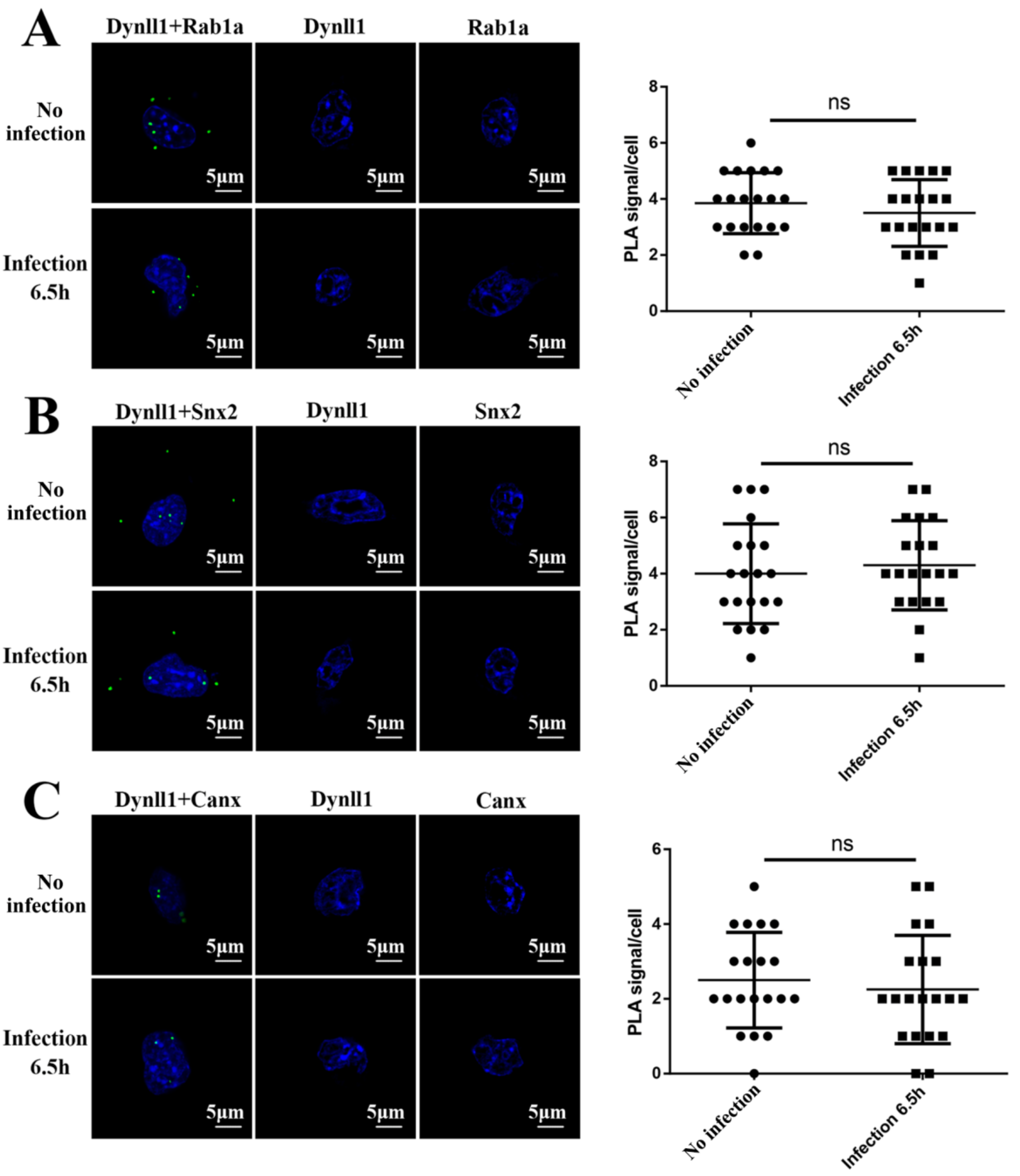

Figure S10 Dynll1 interacting proteins before and after *L. monocytogenes* infection

Dynll1-Rab1a (A), Dynll1-Snx2 (B) and Dynll1-Canx (C) were examined interactions using Proximity ligation assays (PLA) during *L. monocytogenes* infection. Green represents the PLA signal. Two-sided Student's t test was used to measure significance. All data are shown as mean ± SEM.

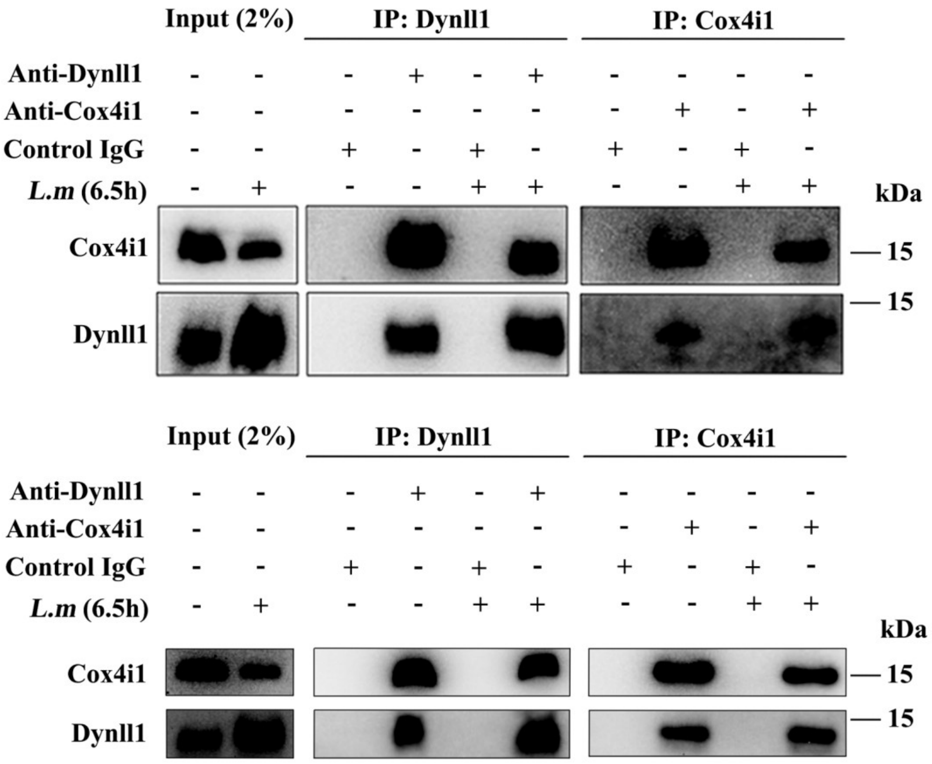

Figure S11 Reduction in the levels of the Dynll1-Cox4i1 complex upon *Listeria* infection was shown by co-IP

The independent co-IP experiments are used to generate the quantitative data for Figure 4D.

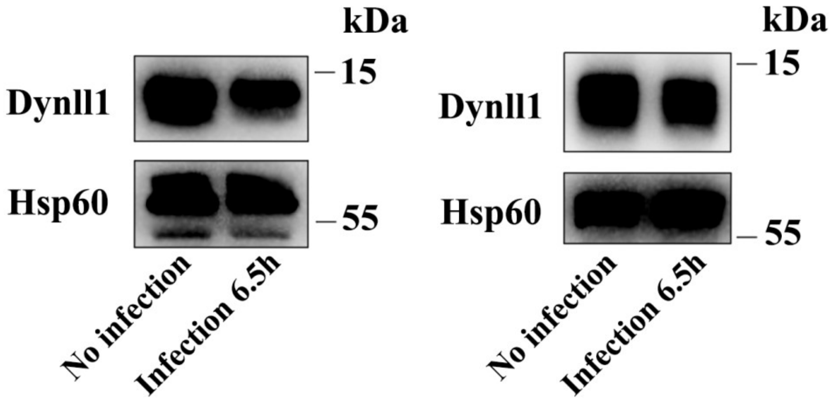

Figure S12 The level of Dynll1 in mitochondria is displayed by western blot upon *Listeria* infection

The independent WB experiments are used to generate the quantitative data for Figure 5D.

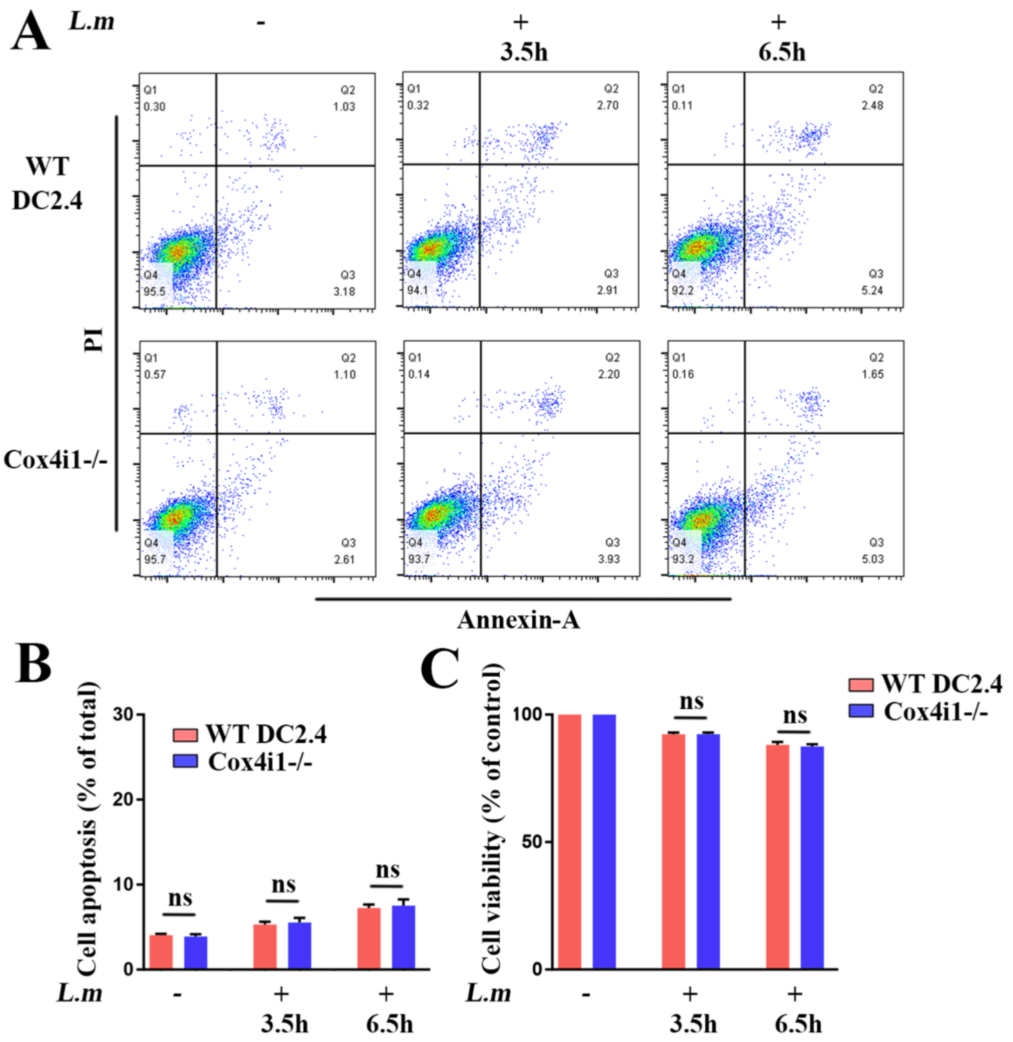

Figure S13 Deletion of Cox4i1 has no associate with host cell death upon *L. monocytogenes* infection

Representative flow cytometric plots (A) and statistical analysis (B) depict apoptosis of WT and Cox4i1-/- mutant DCs upon *L. monocytogenes* infection for 3.5h and 6.5h (n=3). (C) Quantification of living cells of WT and Cox4i1-/- mutant DCs use Cell Counting Kit-8 during *L. monocytogenes* infection for 3.5h and 6.5h (n=3). Two-way ANOVA with Tukey-Kramer tests were used to measure significance. All data are shown as mean ± SEM. * $p < 0.05$, ** $p < 0.01$ and *** $p < 0.001$.

Table S1 Identification of host proteins from membrane proteins mass spectrometry data

Dataset1: A list of 8614 proteins at 1% peptide FDR. Dataset2: A list of 5980 proteins at least one unique peptide and one strict peptide. Dataset3: A list of 4783 protein identified in at least 3 out of 6 experiments. Dataset4: A list of 550 differentially expressed proteins and their values.

Table S2 Identification of *Listeria* proteins from membrane proteins mass spectrometry data

Dataset1: A list of 702 proteins at 1% peptide FDR. Dataset2: A list of 473 proteins with at least one unique peptide and one strict peptide.

Table S3 Identification of Dynll1 interacting proteins from CO-IP mass spectrometry data

Dataset1: A list of 158 proteins at 1% peptide FDR. Dataset2: A list of 158 proteins with at least one unique peptide and one strict peptide. Dataset3: A list of 40 differentially expressed proteins and their values.
